# Vaterschaft und Abhängigkeitserkrankungen – Literaturübersicht qualitativer und quantitativer Studien

**DOI:** 10.1007/s00115-025-01925-4

**Published:** 2025-11-28

**Authors:** Christoph Beineke, Thomas Altenhöner, Janina Dyba, Michael Klein, Thorsten Köhler

**Affiliations:** 1 Fachbereich Sozialwesen, Hochschule Bielefeld, Interaktion 1, 33619 Bielefeld, Deutschland; 2 Fachverband Sucht+ e. V., Bonn, Deutschland; 3 Institut für interdisziplinäre Sucht- und Drogenforschung, Hamburg, Deutschland; 4 Deutsches Institut für Sucht- und Präventionsforschung, Katholische Hochschule Nordrhein-Westfalen, Köln, Deutschland

**Keywords:** Abstinenz, Elternschaft, Familie, Substanzkonsum, Konsumstörung, Abstinence, Parenthood, Family, Substance use, Substance use disorder

## Abstract

**Hintergrund:**

In Angeboten der deutschen Suchthilfe sind ca. 40–50 % der Männer Väter. Betroffene weisen häufig Schwierigkeiten bei der Umsetzung einer gelingenden Vaterschaft auf. Aufgrund daraus hervorgehender Anforderungen kann es zu Konsumänderungen kommen. Eine regelhafte Thematisierung der Vaterschaft im Zuge der Suchtbehandlung wird aktuell nicht umgesetzt.

**Material und Methoden:**

Ziel der narrativen Übersichtsarbeit ist die Darstellung bestehender Zusammenhänge zwischen Suchterkrankungen und einer Vaterschaft. Anhand einer strukturierten Literaturrecherche, durchgeführt im März/April 2023, wurden qualitative und quantitative Erhebungen identifiziert. Eingeschlossen wurden deutsch- und englischsprachige Studien aus den Jahren 2013 bis 2023.

**Ergebnisse:**

Es wurden 20 Studien in die Übersicht eingeschlossen. Suchtkranke Väter sind neben konsumbedingten auch durch väterspezifische Faktoren beeinflusst. Häufig können Betroffene ihre Vaterschaft nur unzureichend und nicht ihren Ansprüchen entsprechend ausleben. Gleichzeitig bietet die Vaterschaft einen motivationalen Antrieb zu Konsumänderungen, wenn sich Väter der negativen Auswirkungen bewusst sind.

**Diskussion:**

Die Daten verweisen auf Wechselwirkungen zwischen einer Abhängigkeit und einer Vaterschaft. Eine gemeinsame Thematisierung in der Suchtbehandlung erscheint sinnvoll, um vorhandenen Bedürfnissen gerecht zu werden. Davon profitieren nicht nur die Väter, sondern auch ihre Kinder.

**Zusatzmaterial online:**

Zusätzliche Informationen sind in der Online-Version dieses Artikels (https://doi.org/10.1007/s00115-025-01925-4) enthalten.

## Abstract (en)

**Background:**

In German addiction services around 40–50% of the men are fathers. Affected fathers often have difficulties in successfully realizing the fatherhood. The resulting demands can lead to changes in consumption; however, fatherhood is currently not regularly addressed as part of addiction treatment.

**Material and methods:**

The aim of this narrative review is to describe existing associations between addictive disorders and fatherhood. A structured literature search conducted in March/April 2023, was used to identify qualitative and quantitative studies. German and English language articles published in 2013–2023 were included.

**Results:**

A total of 20 studies were included in the review. In addition to consumption-related problems, fathers with addictive disorders are also influenced by father-specific factors. Those affected can often only live out their fatherhood insufficiently and not according to their own ideals; however, many fathers are motivated for change if they are aware of the negative effects.

**Conclusion:**

The data point to interactions between addictive disorders and fatherhood. A joint treatment approach can be useful in order to meet existing needs. Not only the fathers can benefit from this but also their children.

Aus einer Abhängigkeitserkrankung können sich Auswirkungen auf die Vaterschaft sowie mitbetroffene Kinder ergeben. Gleichzeitig können aus der Vaterschaft substanzielle Implikationen für den Sucht(hilfe)verlauf entstehen, sowohl hinsichtlich potenzieller Risiken für Konsumverstärkungen als auch hinsichtlich der Änderungsmotivation. Allerdings werden in Deutschland kaum zielgruppenspezifische Angebote vorgehalten. Anhand einer narrativen Übersichtsdarstellung sollen Wechselwirkungen zwischen einer Vaterschaft und einer Konsumstörung aufgezeigt werden.

## Wechselwirkungen zwischen Vaterschaft und Abhängigkeitserkrankungen

### Psychosoziale Problemlagen

Verbunden mit einem problematischen Alltag sehen sich Väter mit einer Drogenkonsumstörung oft nicht in der Lage, ihre Vaterschaft gelingend auszugestalten [3]. Teilweise ist die Vaterschaft trotz generellem Kinderwunsch ungeplant und wird als Schock erlebt [3, 19]. Dabei besteht im Kontext einer Substanzkonsumstörung eine erhöhte Wahrscheinlichkeit, gerade bei Vorfallen häuslicher Gewalt, kein Sorgerecht zu haben und Besuchskontakte gerichtlich zu klären [1, 14, 26]. Im Bereich des problematischen Glücksspiels zeigt eine quantitative Längsschnitterhebung auf, dass Betroffene häufiger eine frühe Vaterschaft erleben und Zusammenhänge mit einem vorzeitigen Ausscheiden aus der Schule, einem niedrigen sozioökonomischen Status, Substanzkonsum sowie Verhaltensstörungen bestehen [13].

In Interviews mit alkoholkonsumierenden Vätern in Kenia berichteten fast alle Befragten von negativen Erfahrungen infolge des Konsums, z. B. die Ausgabe des gesamten Lohns für Alkohol oder körperliche Auseinandersetzungen [18]. Im familiären Kontext zeigen sich finanzielle, emotionale und funktionale Schwierigkeiten [18]. Substanzkonsumstörungen sind weiterhin assoziiert mit psychiatrischen Symptomen, einer problematischen Emotionsregulation, Impulsivität, Schwierigkeiten in Erwachsenenbeziehungen [26], somatischen Folgen des Konsums [1] oder sozialer Isolation [14, 18]. Die materielle Versorgung der Familie gestaltet sich schwierig [14, 18], wobei das Unvermögen, die Ernährerrolle auszuüben, Rollenkonflikte bedingen kann [3]. Aufgrund des Konsums erleben einige Väter [19] und Familienangehörige [18] Stigmatisierung. Ein Teil der Väter berichtet zudem von einer Einbindung in kriminelle Netzwerke [1, 14], wobei einige Kinder polizeiliche Interventionen miterlebten [19, 26].

### Vorstellungen über die Vaterschaft

Während in Interviews mit Vätern mit Substanzkonsumstörung ein eher traditionelles Rollenbild berichtet wird, was die Umsetzung einer involvierten Vaterschaft erschweren kann, werden auch weitere Aufgaben ergänzt [2, 3]. Idealvorstellungen beinhalten z. B. Präsenz, Verantwortung, Offenheit oder Unterstützung, aber auch Autorität und Regulierung [3, 5]. Keinesfalls sollte ein Vater absolut autoritär, aggressiv, gleichgültig oder überreglementierend auftreten, sondern angemessen reagieren [5]. Dabei scheinen biografische Erfahrungen bedeutend. Berichtet wird von einem Vater, der nur als „männlicher Ernährer“, als abwesend oder generell negativ wahrgenommen wurde [3, 5]. Dadurch fehlen positive Rollenvorbilder [2, 3, 5, 22], während Idealvorstellungen in Abgrenzung zu der erlebten Vaterschaft formuliert werden, wobei negative Erfahrungen zur Umsetzung einer „besseren“ Vaterschaft motivieren können [2, 3]. Dennoch erkennen einige Väter Parallelen zwischen der erlebten und der nun umgesetzten Vaterschaft [5], während es einem anderen Teil gelingt, trotzdem eine positive Vaterschaft auszuleben [3]. In einer norwegischen Erhebung mit drogenkonsumierenden Vätern wurden aus den Aussagen drei Vaterbilder identifiziert, die darstellen, wie sich Betroffene als Väter wahrnehmen: der gute, der schlechte und der unsichtbare Vater [22]. Die meisten der acht Teilnehmer erleben sich als unsichtbar und in ihrer Vaterrolle unerwünscht und wertlos [22].

Trotz vorhandener Schwierigkeiten berichten interviewte Väter auch von einer Freude aufgrund ihrer Vaterschaft [3, 5, 19] und es bietet sich eine Möglichkeit zur Umsetzung sozialer Normalitätsvorstellungen im Gegensatz zur stigmatisierten Rolle als abhängige Person [3].

### Beziehung zur Kindesmutter

Die Beziehung zur Kindesmutter gestaltet sich häufig konfliktreich [19], wobei familiäre Beziehungen und Vertrauen konsumbedingt negativ beeinflusst sind [5, 18]. Einige Väter erleben die Androhung von Kontaktverboten bei fortgesetztem Substanzkonsum oder in Konfliktsituationen [2, 22], sodass der Beziehung zur Kindesmutter eine relevante Funktion zur Regulierung des Vater-Kind-Kontaktes zukommt [2, 3]. Für betroffene Väter können sich Ohnmachtsgefühle und Unsicherheiten über ihre Rechte ergeben [19].

Zudem besteht im Kontext von Substanzkonsum ein Risiko für Vorfälle häuslicher Gewalt [2, 14, 18]. In einer quantitativen Befragung zeigt sich, dass Väter, die zu Aggressivität/Feindseligkeit neigen und stark konsumieren, verstärkt physische/psychische Gewalt in der Paarbeziehung und negative Erziehungsstrategien anwenden [28]. Dabei kann sowohl der Substanzkonsum als auch die Aggressivität/Feindseligkeit als Mediator für das jeweils andere Merkmal fungieren [28]. Für betroffene Väter erklärt der Substanzkonsum die Gewalt nicht, gilt aber als verstärkender Faktor in einem Kontext, in dem der Konsum bereits zu Schwierigkeiten in der Beziehung führte [14].

### Umsetzung der Vaterschaft

Substanzkonsum kann dazu beitragen, dass die Umsetzung einer involvierten Vaterrolle nicht gelingt [5, 22] und Väter ihren Ansprüchen nicht gerecht werden [2, 3]. Neben materiellen sind auch emotionale Aspekte eingeschränkt [2], bspw. in Form einer verringerten Empathie, Aufmerksamkeit, Zuwendung, Geduld sowie Schwierigkeiten in Erziehung und Betreuung [3, 14]. Übermäßiger Alkoholkonsum wird einer quantitativen Erhebung zufolge mit einer geringeren Involviertheit assoziiert, wobei dies nicht für alle Länder, in denen die Erhebung umgesetzt wurde, galt [12]. Gerade Drogenintoxikation [3, 22] und Entzugssymptomatik [19, 22] sind durch Verhaltensänderungen geprägt. In Zusammenhang mit Substanzkonsum zeigen sich eine beeinträchtigte Vater-Kind-Beziehung sowie Distanzierungstendenzen der Kinder [3, 5, 18]. Einige Väter haben erlebt, dass ihr Drogenkonsum von älteren Kindern nicht akzeptiert wird, und versuchen ihn vor jüngeren Kindern zu verheimlichen [5], während andere Väter das Bedürfnis äußern, ihre Erkrankung mit dem Kind zu thematisieren [19].

In einer quantitativen Erhebung wurde festgestellt, dass Substanzkonsum mit der Fähigkeit zusammenhängt, die Handlungen anderer als Folge emotionaler Zustände zu verstehen („reflective functioning“, RF) und adäquat zu reagieren [27]. Höheres RF korreliert mit niedrigerem Konsum sowie einer konsistenteren Anwendung disziplinierender Maßnahmen in der Erziehung [27]. Weiterhin ist das väterliche Wissen über das Kind und seine Freizeitgestaltung bei stärkerem Konsum illegaler Substanzen geringer ausgeprägt, während keine Assoziation mit dem Missbrauch von Alkohol oder verschreibungspflichtigen Opioiden bestand [9]. Im Vergleich zwischen Vätern mit und ohne Alkoholabhängigkeit zeigt sich, dass alkoholabhängige Väter signifikant häufiger unsichere Bindungsstile aufweisen [11].

Im Kontext von Substanzkonsum und Gewalt in der Paarbeziehung gilt die Schwere des väterlichen Konsums als Prädiktor für kindliche Vermeidungstendenzen sowie Anspannung in der Beziehung [25]. In einer weiteren Erhebung wurde ein Zusammenhang mit einer geringeren Anwendung positiver, jedoch kein direkter Zusammenhang mit der Anwendung negativer Erziehungsstrategien festgestellt [26].

Problematisches Videospielverhalten steht einer quantitativen Erhebung zufolge in direkter Verbindung mit einer geringeren wahrgenommenen elterlichen Effizienz und depressiven Symptomen [23]. Dabei wurden depressive Symptome als Mediator zwischen dem Videospielen und dem Kompetenzerleben, den Auswirkungen der Elternschaft sowie dem elterlichen Stress beschrieben [23].

Zudem berichten Väter mit Substanzkonsumstörung in mehreren Erhebungen von einer emotionalen oder physischen Abwesenheit [3, 5, 18, 19, 29]. Gründe für die Abwesenheit können Trennungen der Eltern, Kontaktverbote, Haft- oder Therapieaufenthalte sein [2, 3, 14, 22, 26]. Einige Väter gaben an, gerichtliche Auseinandersetzungen zu meiden, jedoch für den Kontakt bereitzustehen, falls die Kinder dies wünschen [19].

### Emotionale Auswirkungen

In Interviews mit drogenkonsumierenden Vätern werden Sorgen darüber berichtet, dem eigenen Kind zu schaden, es einem Risiko zur Entwicklung einer Abhängigkeitserkrankung oder einer Stigmatisierung auszusetzen [3, 19]. Zudem bestehen Sorgen vor nachteiligen Auswirkungen der sozioökonomischen Lebenssituation oder des eigenen Verhaltens auf das Kind [3] sowie die Sorge, den Anforderungen der Vaterschaft aufgrund des Konsums nicht gewachsen zu sein [19]. Im Kontext der Wahrnehmung, eigene Idealvorstellungen aufgrund des Substanzkonsums nicht umsetzen zu können, werden Schuld- und Schamgefühle beschrieben [5, 14, 18, 19]. Einige Väter erleben sich zudem als unerwünscht und von familiären Entscheidungen ausgeschlossen [18, 22]. Ein geringer Kontakt zum Kind kann Stress, Trauer oder Sehnsucht fördern, gleichzeitig können Besuchskontakte mit Druck verbunden sein [19].

### Konsumverstärkungen

Eine als nicht gelingend erlebte Vaterschaft kann im Kontext einer bestehenden Abhängigkeit konsumverstärkend wirken, wenn der Alltag durch besondere Belastungen wie Überforderung, Gewalt oder negative Gefühle geprägt ist [3]. Im Umgang mit entsprechenden Situationen gilt der Substanzkonsum als zentrale Bewältigungsstrategie [18]. In einer Erhebung hing der wahrgenommene Stress mit dem Substanzkonsum zusammen und galt als Moderator zwischen der Fähigkeit zum Emotionsausdruck und dem Substanzkonsum [7]. In einer weiteren Erhebung wurde festgestellt, dass der erlebte, kontextuelle Stress als Moderator zwischen Vaterschaftsstatus und problematischem Konsum einzustufen ist: Bei hohem Stresserleben hingen Vaterschaft und problematischer Konsum signifikant zusammen [6].

### Ressourcen im Kontext der Vaterschaft

#### Konsumänderungen

Die Geburt eines Kindes stellt einen starken Motivator zur Konsumreduktion dar [3, 22], aber auch zu einem späteren Zeitpunkt kann die Erkenntnis einer nichtgelingenden Vaterschaft diese Änderungsmotivation unterstützen [18, 19]. Kontakte zum Kind bieten dabei emotionalen Rückhalt und können tagesstrukturierend wirken [3]. Durch eine ausgeübte Vaterschaft ergeben sich Möglichkeiten zur Stärkung des Selbstwertgefühls sowie des Selbstverständnisses und eine Chance zur Stabilisierung der psychosozialen Situation [3].

Eine Behandlung der Abhängigkeit sehen befragte Väter als Voraussetzung, um die Beziehung zum Kind wechselseitig gestalten zu können, gleichzeitig wird jedoch auch die Bedeutung der Selbstmotivation hervorgehoben [1].

#### Soziale Unterstützung

In einer Befragung von Vätern, die aktuell aufgrund ihrer Opioidabhängigkeit behandelt werden, kommt dem familiären Rückhalt eine hohe Bedeutung zu. Hervorgehoben wird dabei insbesondere der Rückhalt durch die eigene sowie die Kindesmutter [1]. Darüber hinaus berichten befragte Väter, dass sie sich verstärkt auch an andere Personen wenden, um Unterstützung zu erhalten [1]. Die Bedeutung der sozialen Unterstützung durch die eigenen Eltern [18, 19] sowie durch die Partnerin [3, 18] und das weitere Umfeld [18] wird auch in anderen Interviews angeführt.

## Diskussion

Zwischen einer Vaterschaft und einer Abhängigkeitserkrankung bestehen weitreichende Wechselwirkungen. In qualitativen Erhebungen äußern betroffene Väter häufig konkrete und in erster Linie negative sowie durch biografische Erfahrungen geprägte Vorstellungen darüber, wie ein Vater nicht sein sollte. Gleichzeitig bestehen auch positive Erwartungen und Idealvorstellungen. Die Interviewbefunde weisen darauf, dass suchterkrankte Väter diese Ansprüche häufig nicht realisieren können und stattdessen Verhaltensweisen wiederholen, die sie bei ihren eigenen Vätern negativ bewertet haben. Dadurch wird eine Inkongruenz zwischen idealer und umgesetzter Vaterschaft erlebt, wodurch das Risiko für negative Gefühle sowie weitere psychische Belastungen steigt. Anhand der berichteten Ergebnisse zeigt sich, dass suchtkranke Väter sowohl von konsum- als auch von väterspezifischen Problemlagen betroffen sind. Dabei werden Wechselwirkungen zwischen Vaterschaft und Abhängigkeit in beiderlei Richtung sichtbar: Stress, Scham oder Überforderung aus der Situation als Vater können den Konsum verstärken, ebenso sind negative Auswirkungen des Konsums auf die Vaterschaft feststellbar, durch die auch Kinder mitbetroffen sind. In Zusammenhang mit den vorhandenen Problemlagen betroffener Väter besteht die Gefahr, die Thematik stark defizitorientiert zu betrachten und vorhandene Ressourcen zu vernachlässigen. Dabei kann die gelingende Vaterschaft eine positive Zielvorstellung und damit ein motivationales Potenzial für Konsumänderungen bieten. In der Summe zeigen sich in den vorgestellten Erhebungen Hinweise darauf, dass eine Suchterkrankung eine involvierte Vaterrolle erschwert oder verhindert. Das Erleben einer nichtgelingenden Umsetzung der Vaterschaft stellt einen Risikofaktor zur Verschärfung einer bestehenden Konsumproblematik dar. Selbst wenn sich betroffene Väter dieser Einschränkungen bewusst sind, mangelt es oftmals an Ressourcen bzw. Kompetenzen, dies nachhaltig zu ändern. Die Beachtung vaterschaftsbezogener Aspekte im Rahmen der Suchtbehandlung kann dazu beitragen, unzureichende Fähigkeiten im Umgang mit spezifischen Anforderungen zu verbessern, um Konsumsteigerungen als Bewältigungsstrategie vorzubeugen und väterspezifische Faktoren zu fördern [17]. Gerade diese Erkenntnis in Verknüpfung mit einer grundsätzlichen Änderungsmotivation kann einen nachhaltigen Ansatzpunkt für zielgruppenorientierte Unterstützungsangebote im Rahmen der therapeutischen Arbeit bieten. Neben den Eltern können auch Kinder von der Reduktion vorhandener Risiken sowie der Verbesserung ihres Entwicklungsumfeldes profitieren [3, 16].

Allerdings stammen diese Erkenntnisse aus Studien zu substanzkonsumierenden Vätern. Lediglich zwei der Erhebungen [13, 23] beziehen sich auf einen stoffungebundenen Konsum. Wenngleich die Ergebnisse darauf verweisen, dass nachteilige Zusammenhänge mit der Vaterschaft bestehen, sind zentrale Unterschiede zum Substanzkonsum festzuhalten, insbesondere eine fehlende Intoxikation. Direkte Vergleiche vaterschaftsbezogener Implikationen mit stoffgebundenen Problematiken sind aufgrund der limitierten Befundlage derzeit nicht möglich. Wie betroffene Väter ihre Vaterrolle und potenzielle Wechselwirkungen zwischen Spielteilnahme und Vaterschaft interpretieren, bleibt damit noch unklar. Mit Blick auf den Substanzbereich beziehen sich drei Erhebungen [11, 12, 18] ausschließlich auf Alkohol. Vorhandene Erkenntnisse einer narrativen Übersicht aus dem Jahr 2017 verweisen auf substanzspezifische Differenzen im Verhalten abhängigkeitserkrankter Eltern sowie unterschiedliche Zusammenhänge mit psychosozialen Faktoren (im Kontext einer Drogenproblematik z. B. häufigere Abhängigkeit beider Elternteile, stärkere justizielle Probleme, stärkere Auffälligkeit der Kinder, häufigere Trennungen vom Kind) [16]. Dies bezieht sich allerdings nicht konkret auf Väter, sondern auf beide Elternteile. Vor dem Hintergrund der höheren Konsumprävalenzen unter Männern ist dennoch zu vermuten, dass diese Verhaltensweisen eher von den Vätern ausgehen. Eine recht frühe Studie aus dem Jahr 2004, in der Väter mit Substanzkonsumstörung beachtet wurden, berichtet Assoziationen, denen zufolge eine Drogenproblematik mit stärkeren negativen Auswirkungen auf das Erziehungsverhalten der Väter und das Entwicklungsumfeld mitbetroffener Kinder einhergeht, als dies im Kontext einer Alkoholproblematik feststellbar war [10]. Der Frage nach differenten Wechselwirkungen verschiedener Konsumformen mit der Vaterschaft, gerade auch mit Blick auf stoffgebundene und stoffungebundene Problematiken, sollte in zukünftigen Studien noch einmal spezifisch nachgegangen werden. Anhand der eingeschlossenen Studien in der vorliegenden Übersicht ist ein derartiger Gruppenvergleich nicht möglich. Es zeigen sich für die verschiedenen Konsumformen Wechselwirkungen mit der Vaterschaft, die weit überwiegend negativ ausfallen, ohne dass Unterschiede zwischen den Konsumformen sichtbar werden.

In Kongruenz mit den dargestellten Erkenntnissen wird in mehreren Publikationen die Entwicklung väterspezifischer Angebote gefordert [3, 5, 16, 22]. Insbesondere in den USA wurden erste entsprechende Programme implementiert, die sich spezifisch an Väter richten [15, 24]. In Deutschland sind bisher keine spezifischen Väterprogramme veröffentlicht, einen Ansatzpunkt bietet lediglich das Handbuch *Männlichkeiten und Sucht* [4]. Demgegenüber existieren einzelne Interventionen, die sich generell an Eltern oder speziell an Mütter richten, ebenso wie Interventionen für Kinder aus suchtbelasteten Familien [16]. Weiterhin sollte die Vaterschaft auch als Thema innerhalb bestehender Angebote berücksichtigt werden.

## Fazit für die Praxis

- Zwischen einer Vaterschaft und Abhängigkeitserkrankungen bestehen zahlreiche Wechselwirkungen, sodass die Vaterschaft einen zentralen Stressor im Alltag ebenso wie eine zentrale Ressource im Suchthilfeverlauf darstellen kann.
- Die Umsetzung einer gelingenden Vaterschaft kann eine zentrale Motivation zu Behandlungsaufnahme und -abschluss darstellen – dies sollte wertschätzend aufgegriffen und gefördert werden.
- Neben dem Rückgriff auf bestehende, evidenzbasierte Elterninterventionen sollten väterspezifische Angebote entwickelt, implementiert und evaluiert werden.
- Von einer Beachtung der Vaterschaft im Rahmen der Suchthilfe können nicht nur Väter, sondern auch mitbetroffene Kinder profitieren.

## Supplementary Information

Online-Tabelle 1: Übersicht der eingeschlossenen Studien

Hintergrund und Methoden
