# Supplementary material for: Fatherhood and addiction disorders—Literature review of qualitative and quantitative studies
Source: Nervenarzt. 2025 Nov 28;97(6):590–4. [Article in German] doi: 10.1007/s00115-025-01925-4 (PMC13719710; doi:10.1007/s00115-025-01925-4)
Supplement: Supplementary file 1 [file 115_2025_1925_MOESM1_ESM.pdf]

Online-Tabelle 1: Übersicht der eingeschlossenen Studien

**Studie**	**Spezifikation der Stichprobe (N und Alter) und Einschlusskriterien**	**Beachteter Konsum**	**Erhebungs-land**	**Methodik Rekrutierung**	**Themen der Erhebung**
Bakos-Block, Nash, Cohen et al. (2022) [1]	N=14 (3 Väter, 11 Mütter)<br>Alter: 27-54 Jahre<br><br>*Einschlusskriterien:*<br>• ≥ 18 Jahre<br>• Minderjähriges Kind während der Abhängigkeit und dem Beginn einer Unterstützungsmaßnahme<br>• Aktuelle Behandlung	Opioide	USA	Qualitativ: Einzelinterviews (leitfadengestützt, semi-strukturiert)<br><br>*Rekrutierung:* Bereitstellung von Informationen in einer Selbsthilfegruppe, Interessierte konnten sich selbst melden	Barrieren zur Inanspruchnahme von Unterstützung, Ressourcen, Motivation
Benoit & Magnus (2017) [2]	N=34 (8 Väter, 26 Mütter)<br>Alter: Median Väter: 26 Jahre, Median Mütter: 30 Jahre<br><br>*Einschlusskriterien:*<br>• ≥ 19 Jahre<br>• Direkte oder indirekte Beeinflussung durch Substanzkonsum<br>• Niedriges Einkommen oder unsichere Wohnverhältnisse<br>• Schwangerschaft/schwangere Partnerin oder Geburt in den letzten 12 Monaten	Alkohol, illegale Drogen	Kanada	Qualitativ: Einzelinterviews<br><br>*Rekrutierung:* Ansprache über Poster in öffentlichen Einrichtungen (sozial, Gesundheitsservice), Vorschläge von Fachkräften und Teilnehmenden für weitere potenzielle Kandidat_innen	Vaterrolle, Unterschiede in der Beschreibung problematischen Substanzkonsums zwischen Vätern und Müttern, Einbindung von Vätern in ein spezifisches Programm für Mütter, die durch Substanzkonsum, Gewalt oder Mental Health Probleme beeinflusst sind, Thematisierung des Konsums zwischen den Elternteilen
Bernard & Tödte (2017) [3]	N=24<br>Alter: M=37 Jahre<br><br>*Einschlusskriterien:*<br>• Konsum illegaler Substanzen<br>• Mind. ein leibliches Kind <18 Jahre	Illegale Substanzen	Deutschland	Qualitativ: Einzelinterviews (leitfadengestützt)	Vaterbilder und -konzepte, Ausübung der Vaterschaft, Wechselwirkungen zwischen Vaterschaft und Drogenkonsum, Herausforderungen/Belastungen, Hilfe-/Unterstützungsbedarfe
Caponnetto, Triscari & Maglia (2020) [5]	N=18<br>Alter: 21-56 Jahre, M=42,3 Jahre, Median: 38,5 Jahre	Heroin, Kokain	Italien	Qualitativ: Einzelinterviews (semi-strukturiert)<br><br>*Rekrutierung:* Ansprache in Einrichtung der Suchthilfe, Kombination von gezieltem und Quotensampling	Familiärer Kontext, Vertrauen in Partner_in, Beziehung zum eigenen Vater und Abwesenheit einer Vaterfigur, Selbstwahrnehmung als Vater, Idealvorstellungen über die Vaterrolle, Auswirkungen der Abhängigkeit auf die Vater-Kind-Beziehung

	Einschlusskriterien:<br>• Substanzkonsumstörung (Dauer ≥ 6 Monate)<br>• Vater in stationärer Behandlung<br>• Kein Vorliegen einer Major Depression oder anderer psychiatrischen Störungen				
Curtis, Collins, Augustine et al. (2022) [6]	N=476, davon 116 Väter<br>Alter: 19-22 Jahre, M=20,3 Jahre<br><br>*Einschlusskriterien:*<br>• Selbstidentifikation als „Black American“ oder „African American“<br>• Wohnort im Erhebungsgebiet<br>• Männliches Geschlecht<br>• Alter zwischen 19 und 22 Jahren<br>• Unverheiratet	Zigaretten, Alkohol, Cannabis, andere Drogen	USA	Quantitativ: Befragung (längsschnittlich)<br><br>*Rekrutierung:* Über 45 initiale Teilnehmer, die jeweils weitere potenzielle Teilnehmer vorschlugen, bis eine Stichprobengröße von N ≥ 500 erreicht wurde	Einfluss des Stresserlebens auf den Zusammenhang zwischen Vaterschaft und Substanzmissbrauch
Desrosiers, Sipsma, Divney et al. (2015) [7]	N=380 (175 Väter, 205 Mütter)<br>Alter: $M_{Väter}$=21,3 Jahre, $M_{Mütter}$=18,7 Jahre<br><br>*Einschlusskriterien:*<br>• Schwangerschaft (der Partnerin) im 2. oder 3. Trimester<br>• Werdende Mütter (14-21 Jahre), werdende Väter (≥14 Jahre)<br>• Feste Beziehung des Paares<br>• Leibliche Elternschaft beider Eltern<br>• Einverständnis zur Teilnahme<br>• Englische oder spanische Sprachkenntnisse	Alkohol, Zigaretten, Cannabis	USA	Quantitativ: Befragung (längsschnittlich)<br><br>*Rekrutierung:* Ansprache über Kliniken für Geburtshilfe und Gynäkologie	Assoziation zwischen emotionaler Ausdruckfähigkeit und Substanzkonsum, Analyse der Mediatoren Depression und Stress in Bezug auf die Assoziation

Elam, Sternberg, Waddell et al. (2020) [9]	N=825 (368 Väter, 457 Mütter)<br>Alter: $M_{Väter}$=35,9 Jahre, $M_{Mütter}$=34,6 Jahre	Alkohol, verschreibungs-pflichtige Opioide	USA	Quantitativ: Befragung (längsschnittlich)<br><br>*Rekrutierung:* Ansprache über Telefonscreenings, Gerichtsakten und Gesundheitsdienste	Zusammenhänge zwischen elterlicher Alkoholabhängigkeit, Opioidmissbrauch und elterlichem Wissen über Freizeitaktivitäten des Kindes im Jugendalter, Determinanten zukünftigen Alkoholkonsums des Kindes, Einfluss elterlichen Wissens als Mediator zwischen elterlichem und kindlichem Konsum
Hazarika & Bhagabati (2018) [11]	N=200 (je n=50 Väter mit und ohne Alkoholabhängigkeit sowie Söhne mit und ohne alkoholabhängigen Vater)<br><br>*Einschlusskriterien:*<br>• Mittleres Alter (40-60 Jahre)<br>• Mittlerer sozioökonomischer Status<br>• Nicht stammeszugehörig<br>• Abschluss der High School<br>• Alkoholabhängigkeit (Ergebnis von ≥ 13 Punkten im Michigan Alcoholism Screening Test)<br>• Söhne: ≥15 Jahre mit Abschluss der High School	Alkohol	Indien	Quantitativ: Befragung (Fall-Kontroll-Studie)<br><br>*Rekrutierung:* Ansprache über Suchthilfeeinrichtungen (alkoholabhängige Väter), Schneeballverfahren (unbelastete Väter)	Bindungsstile von Vätern mit und ohne Alkoholabhängigkeit sowie ihrer Söhne
Laslett, Kuntsche, Wilson et al. (2022) [12]	N=4.562<br>Alter: 18-49 Jahre	Alkohol (Rauschtrinken)	Kambodscha, China, Indonesien, Papua-Neuguinea, Sri Lanka	Quantitativ: Befragung (querschnittlich)<br><br>*Rekrutierung:* Zufallsstichprobe für jedes Land aus ausgewählten repräsentativen Gebieten	Zusammenhang zwischen väterlichem Rauschtrinken und Erziehungsbeteiligung, Überprüfung erlebter Kindheitstraumata als Moderator in diesem Zusammenhang
Lee, Storr, Ialongo et al. (2013) [13]	N=294, davon 190 Glücksspieler und 120 junge Väter<br><br>*Einschlusskriterien:*<br>• Vorliegende Daten zu Vaterschaft und Glücksspielverhalten in der Hauptstudie (Teilnahme an Follow-Up-Erhebungen im Alter von 17-22 Jahren)	Glücksspiel	USA	Quantitativ: Befragung (längsschnittlich)<br><br>*Rekrutierung:* Nutzung vorliegender Daten einer Studie, die in neun verschiedenen Schulen umgesetzt wurde, um eine in der ersten Klasse durchgeführte Intervention zu evaluieren	Zusammenhang von Glücksspielverhalten und junger Vaterschaft (im Alter von 20 Jahren)

Lessard, Lévesque, Lavergne et al. (2021) [14]	N=43 (16 Väter, 15 Mütter, 12 Jugendliche)<br>Alter: $M_{Väter}$=39 Jahre, $M_{Mütter}$=36 Jahre, M_{Kinder}=14 Jahre<br><br>*Einschlusskriterien:*<br>• Mind. ein minderjähriges Kind mit regelmäßigem Kontakt<br>• Mind. eine Situation von häuslicher Gewalt in den letzten zwei Jahren in Verbindung mit anderem elterlichen Problem (mental oder Konsum)	Alkohol, Drogen, Medikamente, Internet	Kanada	Qualitativ: Einzelinterviews (semi-strukturiert)<br><br>*Rekrutierung:* Über Fachkräfte öffentlicher Einrichtungen (Sucht, mentale Gesundheit, häusliche Gewalt), die das Projekt vorstellten und Kontaktdaten von Interessierten weiterleiteten	Erleben der Zusammenhänge von häuslicher Gewalt, Substanzkonsumstörungen und Mental Health Problemen, Vorkommen dieser Probleme im familiären Kontext, Auswirkungen gemeinsam auftretender Probleme auf Elternschaft bzw. Erziehung, Eltern-Kind-Beziehung und persönliches Wohlbefinden
Patel, Kaiser, Meade et al. (2020) [18]	N=31 (11 Väter, 11 Mütter, 9 Kinder)<br>Alter: $M_{Väter}$=38 Jahre, $M_{Mütter}$=32 Jahre, M_{Kinder}=12 Jahre<br><br>*Einschlusskriterien:*<br>• Erziehungsverantwortung für ein 8- bis 17-jähriges Kind<br>• Gemeinsam lebend mit der Familie<br>• Problematischer Alkoholkonsum (AUDIT-Score 3-19 Punkte)	Alkohol	Kenia	Qualitativ: Einzelinterviews (leitfadengestützt, semi-strukturiert)<br><br>*Rekrutierung:* Ansprache über Pastoren und Gemeindeleiter	Erfahrene familiäre/organisationale Unterstützung, Rolle der Familie/sozialer Netzwerke als Hindernis/Unterstützung zur Inanspruchnahme, Wahrnehmung des Alkoholkonsums und der erhaltenen Unterstützung, Auswirkungen des Konsums auf Familie/soziale Netzwerke
Pihkala & Sandlund (2015) [19]	N=12 (7 Väter, 5 Mütter)<br>Alter: Väter: 25-49 Jahre, Median: 33 Jahre<br>Mütter: 29-37 Jahre, Median: 33 Jahre<br><br>*Einschlusskriterien:*<br>• Aktuelle Substitutionstherapie<br>• Minderjähriges Kind<br>• Kontakt zum Kind	Opioide	Schweden	Qualitativ: Einzelinterviews (leitfadengestützt)<br><br>*Rekrutierung:* Ansprache über Fachkräfte der Suchthilfe	Erfahrungen als Eltern, z. B. Schwangerschaft und Geburt, erlebte Unterstützung, Sorgen, Diskriminierungserfahrungen, Ansichten zu Drogen und Elternschaft
Söderström & Skarderud (2013) [22]	N=8<br>Alter: 30-49 Jahre<br><br>*Einschlusskriterien:*<br>• Substanzkonsumstörung (ICD-10)<br>• Kind im Alter von 0-6 Jahren<br>• Norwegisch als Hauptsprache	Illegale Substanzen	Norwegen	Qualitativ: Gruppendiskussionen (gemischt- & gleichgeschlechtlich) In der Studie wurden nur die Aussagen der männlichen Teilnehmer genutzt.<br><br>*Rekrutierung:* Ansprache in Einrichtung der Suchthilfe	Selbst- und Fremdwahrnehmung als Vater, Erstellung von Idealtypen

Stockdale & Coyne (2020) [23]	N=481 Mütter,<br>N=374 zu den Müttern gehörende eingeladene Väter<br>Alter: $M_{Väter}$=32,4 Jahre, $M_{Mütter}$=31,0 Jahre<br><br>Videospielende:<br>Väter: 56%, Mütter: 30%<br><br>*Einschlusskriterien:*<br>• Geburt des Kindes innerhalb des letzten Jahres	Videospiel	USA	Quantitativ: Befragung (querschnittlich)<br><br>*Rekrutierung:* Anschreiben aller Frauen im Großraum Denver, die im vergangenen Jahr Mutter wurden, zusätzlich Ansprache aller zusammenlebender Väter, persönliches Aufsuchen durch im Projekt Beschäftigte, ergänzend Flyer in öffentlichen Einrichtungen	Einfluss von pathologischem Videospiel auf elternschaftsbezogene Variablen (Wirksamkeit, Kompetenz, Auswirkungen der Elternschaft, Stress), depressive Symptome als Mediator
Stover & Coates (2016) [25]	N=24<br>Alter: M=33,8 Jahre<br><br>*Einschlusskriterien:*<br>• Biologische Vaterschaft eines Kindes ≤ 7 Jahre<br>• Mind. monatlicher Kontakt<br>• Substanzmissbrauch (DSM-IV) und Konsum der Substanz in den letzten 60 Tagen<br>• Vorfall häuslicher Gewalt in den letzten 6 Monaten	Alkohol, Cannabis, Opiate	USA	Mixed-Methods: Quantitative Befragung und qualitative Beobachtung (querschnittlich)<br><br>*Rekrutierung:* Ansprache über Flyer/Broschüren in einer Suchthilfeeinrichtung zur Teilnahme an einer Intervention zur Reduktion von Substanzkonsum und häuslicher Gewalt	Zusammenhänge zwischen Erziehungsverhalten, Mentalisierungsfähigkeit (Verständnis & Berücksichtigung mentaler Zustände des Kindes) und Vater-Kind-Interaktionen
Stover, Easton & McMahon (2013) [26]	N=86 (43 Väter mit Substanzmissbrauch und häuslicher Gewalt, 43 unbelastete Väter)<br>Alter: M=34,7 Jahre<br><br>*Einschlusskriterien:*<br>• Alkohol-, Kokain- oder Cannabismissbrauch (DSM-IV)<br>• Konsum in den letzten 30 Tagen<br>• Vorfall häuslicher Gewalt in den letzten 12 Monaten<br>• Mit biologischem Kind (2-6 Jahre) lebend bzw. mind. monatlicher Kontakt	Alkohol, Kokain, Cannabis, weitere illegale Substanzen	USA	Quantitativ: Befragung (Fall-Kontroll-Studie)<br><br>*Rekrutierung:* community-basiert, Flyer in öffentlichen Einrichtungen	Gruppenvergleich von belasteten und unbelasteten Vätern in Bezug auf Substanzkonsum und Anwendung häuslicher Gewalt, Auswirkungen auf und Mediatoren für das Erziehungsverhalten und emotionale/Verhaltensprobleme bei den Kindern

Stover & Kiselica (2014) [27]	N=79 (davon 52,5% mit Substanzmissbrauch und häuslicher Gewalt, 1 Vater nur mit Substanzmissbrauch, 47,5% unbelastet)<br>Alter: 20-52 Jahre, M=34,7 Jahre<br><br>*Einschlusskriterien:*<br>• Biologische Vaterschaft eines Kindes im Alter von 2-6 Jahren<br>• Mind. monatlicher Besuchskontakt	Alkohol, Cannabis, Opiate, Halluzinogene, Kokain	USA	Quantitativ: Befragung (querschnittlich)<br><br>*Rekrutierung:* Flyer in öffentlichen Einrichtungen, Strategie, dass die Stichprobe zu jeweils der Hälfte aus Probanden mit bzw. ohne Substanzmissbrauch/häuslicher Gewalt besteht	Zusammenhänge von elterlicher Mentalisierungsfähigkeit und Erziehungsverhalten, Assoziationen weiterer Faktoren zur Mentalisierungsfähigkeit
Stover & Kiselica (2015) [28]	N=132 (davon 36,80% mit Substanz-missbrauch und häuslicher Gewalt, 8,33% nur häusliche Gewalt, 21,21% nur Substanzmissbrauch, 33,67% unbelastet)<br>Alter: 20-52 Jahre, M=34,6 Jahre<br><br>*Einschlusskriterien:*<br>• Biologischer Vater eines Kindes im Alter von 2-6 Jahren<br>• Mind. monatlicher Kontakt	Alkohol, Cannabis, Opiate, Sedativa, Halluzinogene, Kokain, Inhalanzien, Amphetamine	USA	Quantitativ: Befragung (querschnittlich)<br><br>*Rekrutierung:* Flyer in öffentlichen Einrichtungen, Strategie, dass die Stichprobe mind. zur Hälfte aus Vätern mit Problemen im Bereich Substanzkonsum und/oder häuslicher Gewalt besteht	Zusammenhang zwischen Feindseligkeit und häuslicher Gewalt sowie negativen Erziehungsstrategien, Überprüfung des Substanzkonsums als Mediator in diesem Zusammenhang, Interaktionseffekte von Feindseligkeit und Substanzkonsum
Ward, Kippen, Reupert et al. (2021) [29]	N=744, davon 394 Eltern (233 Väter, 160 Mütter)<br>Alter: $M_{Väter}$=39,1 Jahre, $M_{Mütter}$=35,6 Jahre<br><br>*Einschlusskriterien:*<br>• ≥ 18 Jahre<br>• Mind. monatlicher Methamphetamin-Konsum (primär rauchend) in den vergangenen 6 Monaten<br>• Lebensmittelpunkt in/um Victoria	Methamphetamin	Australien	Quantitativ: Befragung (querschnittlich)<br><br>*Rekrutierung:* Kombination aus Gelegenheitsstichprobe und durch bereits Befragte gesteuertes Schneeballverfahren	Assoziationen zwischen Elternstatus, Zusammenleben des Vaters/der Mutter mit dem Kind und soziodemografischen, psychosozialen, mentalen, konsumbezogenen (Methamphetamin-Abhängigkeit/schädlicher Alkoholkonsum) Faktoren
