# Supplementary material for: Fatherhood and addiction disorders—Literature review of qualitative and quantitative studies
Source: Nervenarzt. 2025 Nov 28;97(6):590–4. [Article in German] doi: 10.1007/s00115-025-01925-4 (PMC13719710; doi:10.1007/s00115-025-01925-4)
Supplement: Supplementary file 2 [file 115_2025_1925_MOESM2_ESM.pdf]

Hintergrund

2021 war in Deutschland jeder vierte Mann im Alter von 18-64 Jahren von einem problematischen Alkoholkonsum betroffen, 3,7% haben illegale Substanzen problematisch konsumiert, wobei Männer höhere Prävalenzen aufweisen [20]. 2023 gaben 39% der ambulant und 50% der stationär behandelten Männer an, ein Kind zu haben, jeweils 26% hatten ein minderjähriges Kind, wobei nur ein geringerer Anteil mit einem Kind zusammenlebte [21]. Valide Daten über die Anzahl abhängigkeitserkrankter Väter liegen allerdings nicht vor.

Mit einem (Substanz-)Konsum werden verschiedene psychosoziale Problemlagen assoziiert, aus denen sich Konsequenzen für den Alltag betroffener Männer und für die Vaterschaft ergeben können. Zu nennen sind dabei bspw. Komorbiditäten mit weiteren psychischen Erkrankungen und eine prekäre ökonomische Situation [16]. Neben allgemeinen Verhaltensänderungen aufgrund des Konsums bestehen Zusammenhänge mit inadäquaten Erziehungsstilen, die sich einerseits in häufigerem Überreagieren, andererseits jedoch auch in zu schwachen, nachlässigen Reaktionen auf kindliche Anforderungen und Fehlverhalten zeigen [8, 16]. Bei mitbetroffenen Kindern zeigen sich emotionale und Verhaltensprobleme sowie Schwierigkeiten im sozialen Miteinander [8] und das Risiko zur Entwicklung einer Abhängigkeits- oder anderen psychiatrischen Erkrankung ist erhöht [16]. Zudem steigt infolge konsumbedingter Problemlagen das Risiko für Kontaktabbrüche zwischen Eltern und Kind [16]. Während sich aus der Elternschaft einerseits eine starke Abstinenzmotivation für abhängigkeitserkrankte Personen ergeben kann, sind andererseits potenzielle Konsumverstärkungen infolge der Anforderungen und Belastungen zu beachten [3].

Dennoch liegt der Fokus in der wissenschaftlichen und praktischen Auseinandersetzung in Deutschland wie auch international überwiegend auf Müttern, während Väter häufig ignoriert werden [3, 24], wozu auch soziale Rollenvorstellungen beitragen [22]. Dadurch fehlen Kenntnisse über vaterschaftsbezogene Aspekte im Kontext der Abhängigkeitserkrankung zur Entwicklung zielgruppenorientierter Angebotsstrukturen [3]. Aufgrund der vorhandenen Wechselwirkungen zwischen Vaterschaft und Abhängigkeitserkrankung können genau solche Angebote jedoch zu einer Verbesserung der Gesamtsituation beitragen [3]. Im Folgenden soll deshalb der Frage nach Wechselwirkungen zwischen Vaterschaft und Abhängigkeitserkrankung nachgegangen werden.

Methodik

Grundlage der Übersicht bildet eine strukturierte Literaturrecherche, in der deutsch- und englischsprachige Publikationen aus dem Zeitraum Januar 2013 bis März 2023 eingeschlossen wurden. Die Literatursuche wurde in den Datenbanken LIVIVO, PubMed, PSYNDEX und Cochrane Library umgesetzt. Ergänzend wurden zwei händisch identifizierte Studien aus anderen Quellen aufgrund ihrer thematischen Relevanz einbezogen. Zur Recherche der Veröffentlichungen wurden die Begriffe *„addiction"*, *„dependence"*, *„substance use"* *„gaming"* und *„gambling"* mit *„father"*, *„fatherhood"*, *„fathering"* und *„paternal"* kombiniert, um die Zielgruppe der suchtkranken Väter zu beschreiben, ohne weitergehende Einschränkungen (z. B. Alter des Kindes) vorzunehmen. In die Übersicht eingeschlossen wurden qualitative und quantitative Erhebungen, in denen Zusammenhänge zwischen einem problematischen Konsum oder einer Abhängigkeit von Alkohol, illegalen Drogen, Glücksspiel und Videospielen und einer Vaterschaft berichtet werden. Anhand der Literaturrecherche wurden 4.760 Publikationen aufgefunden, davon wurden 1.048 Dubletten vor der Durchsicht ausgeschlossen. Die Texte wurden anhand des Titels gescreent, darauf aufbauend wurden 204 Titel tiefergehend anhand des Abstracttextes und davon 38 anhand des Volltextes systematisiert.

Abschließend wurden 20 Texte für die Übersicht ausgewählt, dabei handelt es sich um elf quantitative und acht qualitative Studien sowie eine, die einem Mixed-Methods-Ansatz folgt (Online-Tab. 1). Sechs

Erhebungen beziehen sich auf den Konsum illegaler Substanzen, drei auf den Konsum von Alkohol und jeweils eine auf Glücks- sowie Videospiele. Die restlichen neun Erhebungen beachten verschiedene Konsumgruppen, zumeist Alkohol und illegale Substanzen. Lediglich eine Erhebung beinhaltet neben substanzbezogenen ebenfalls internetbezogene Problematiken. Eine Beurteilung der Studienqualität fand nicht statt. Aus den berücksichtigten Publikationen wurden die wesentlichen Ergebnisse mit Blick auf Zusammenhänge zwischen einer Vaterschaft und einer Abhängigkeitserkrankung in Form einer narrativen Übersicht dargestellt.
